# Annotations

**Published:** 1887-08-13

**Authors:** 


### ANNOTATIONS.

**Costly Patients.**

£200 per bed per annum! Such is the cost of each in-patient at the Swansea Fever Hospital, if "An Indignant Ratepayer" writing to the *Swansea Herald* may be trusted. The managers of the hospital, and if there be no managers, the officials in residence must make haste to answer so serious a charge as this. It is not a case of mismanagement, but of something which goes by a much uglier name if the charge be true. We make it a rule to condemn no institution unheard: but if there be not a prompt acceptance of the challenge thus offered, and an audited statement which will completely refute the accusation, steps must be taken for an inquiry of another kind. "It appears to me," says the *Herald's* correspondent, "that the officials at the Fever Hospital do just as they please, charge just what they please, admit whom they please, and discharge their patients when they please, without reference to any responsible authority." Who are these officials? How have they come by their positions? How long have they been spending the public money at this rate? The town of Swansea owes it to itself that a full and adequate inquiry be made, and that without delay. The expenditure of public money is an honourable trust, and any person who wastes the funds of charity by mismanagement or conduct deserving a still more opprobrious name, must be promptly exposed, and as quickly brought to just and merited punishment.

**Where is the Colchester Physician?**

Colchester is in a condition of retrograde metamorphosis, at least so far as its medical faculty is concerned. The physicianship at the hospital—a venerable institution—has disestablished itself by mere lapse of time. "O tempora: O mores: O dii immortales!" Where is the Gibbon who shall fitly write the history of the "decline and fall" of the Colchester physicianship? The Rev. J. W. Irvine, speaking at the half-yearly meeting, said: "At the annual meeting held in November last, the governors were greatly pressed by the professional staff not to take any steps in the alteration of their rules, in the hope that a physician would be forthcoming. The medical staff assured them they would be very strenuous in their exertions to get one. It was not for him to say whether they had been strenuous or not, but he knew they had been unsuccessful, and at the meeting held the other day, they had been told that the task of getting a physician was quite hopeless." Will the medical men of Colchester now recognise that the times are changed, and that it is necessary for them to change with them? Have they so little historical knowledge, and such very feeble reasoning powers, as to think that Colchester and—still greater absurdity—the medical staff of the Colchester Infirmary is to stem the tide of change in human affairs? Do they ever see *Punch;* and have any of them made the acquaintance of Mrs. Partington and her mop? The governors, at any rate, must see to it that at all risks the efficiency of the hospital is steadily and progressively maintained.

**Doctors in Council.**

Dublin has just witnessed a most impressive medical consultation, which it may be hoped it will survive. Doctors of almost every nation, people, and tongue have been gathered together in the Irish capital, under the auspices of the British Medical Association. The occasion has brought about one of the most significant occurrences of recent times—the practical union of Protestant and Catholic Christians in religious services. For the benefit of the Association, which used to have the credit of being materialistically inclined, the Catholic and Protestant cathedrals had services simultaneously, both of which were exceedingly well attended. The highest dignitaries of both churches took advantage of the occasion to place before their audiences some well-reasoned considerations in favour of that religion which is alike the common possession and the undying hope of all mankind. Not the least of the advantages which may result from the meeting of the British Medical Association in Dublin is this practical, if temporary union of opposing faiths in one great effort to meet science and philosophy, as represented by physic, half-way. Religion can suffer no loss by being allied with science, and the medical art with its scientific ancillaries is always humanised and advantaged by friendly contact with the cultivated representatives of the Christian philosophy and faith.

**Unparalleled Scepticism.**

In his address on Medicine just delivered to the British Medical Association at Dublin, Dr. A. J. Gairdner recalled to the memory of the distinguished practitioners there assembled the sceptical query of the late Sir William Hamilton, published in the *Edinburgh Review* of 1832: "Has the *practice* of medicine (that is, the art as distinguished from the science) made a single step since Hippocrates?" This question, asked with something of a sneer by the illustrious metaphysician, was repeated in sober seriousness by Dr. Warburton Begbie in 1875, and is now again brought before the profession by Dr. Gairdner in the same spirit of earnest philosophic inquiry. What a shock must the question give to many of the boastful young men of the present day, who vainly imagine that "they indeed are the men, and that wisdom will die with them." It is the misfortune of large numbers of youthful "physicians and scientists" that their reading is confined to the books which immediately tell on examinations and present-day controversies. Such reading is necessarily limited, and soon comes to an end. The young man who is "well up" in this thinks he has mastered all knowledge and is complete at every point. The truth is that in many cases he has yet to learn the simplest elements of the philosophy of his science and art. The details he often knows fairly well, as at present practised. The reason and soul of medicine itself he has never entered into. Dr. Gairdner thinks that, on the whole, progress has been made, and that at least the foundations of modern medicine are laid on rock, and not on shifting sand. But that he should venture to propound Sir William Hamilton's question at all before the most learned professors of the physician's art known to the world of to-day is a striking and much needed reproof to those shallow students who think that because they have fished in a mill-pond they must necessarily know all the varieties of animal life which abound in the depths of the great oceans.

